# Profiling Human Papillomavirus Lineage-Specific Capsid Antigenicity With Geographically Diverse Natural Infection Antibodies

**DOI:** 10.1093/infdis/jiaf502

**Published:** 2025-10-01

**Authors:** Kavita Panwar, Kazutomo Yokoya, Sofia Gomes, Vanessa Tenet, John Schussler, Rolando Herrero, Lisa Mirabello, John T Schiller, Mónica S Sierra, Gary M Clifford, Simon Beddows

**Affiliations:** Virus Reference Department, Public Health Microbiology Division, UK Health Security Agency, London, United Kingdom; Virus Reference Department, Public Health Microbiology Division, UK Health Security Agency, London, United Kingdom; Virus Reference Department, Public Health Microbiology Division, UK Health Security Agency, London, United Kingdom; Early Detection, Prevention and Infections Branch, International Agency for Research on Cancer, Lyon, France; Information Management Services Inc, Silver Spring, Maryland; Agencia Costarricense de Investigaciones Biomédicas, formerly Proyecto Epidemiológico Guanacaste, Fundación INCIENSA, San José, Costa Rica; Division of Cancer Epidemiology and Genetics; Center for Cancer Research, National Cancer Institute, Bethesda, Maryland; Division of Cancer Epidemiology and Genetics; Early Detection, Prevention and Infections Branch, International Agency for Research on Cancer, Lyon, France; Virus Reference Department, Public Health Microbiology Division, UK Health Security Agency, London, United Kingdom

**Keywords:** HPV, natural infection, lineage, antigen, antibody

## Abstract

Human papillomavirus variants are classified into lineages based on their whole genome sequence, but the impact of lineage variation on the structure or function of encoded proteins is unclear. We used a global panel of lineage-specific natural infection sera to assess antibody-binding specificity for lineage-specific L1L2 antigens, and we used these data to create relational antigenic maps. Merging these data with neutralizing antibody data demonstrated similar spatial geometry and revealed dependency on a limited number of amino residues on the capsid surface. These data inform the degree of lineage specificity within the natural infection humoral immune response to oncogenic human papillomaviruses.

## Teaser

Human papillomavirus lineages display differential phylogeographic distribution, but little is known about the impact of variation on the structure and function of encoded proteins. Relational antigenic maps informed the lineage-specific antigenicity of oncogenic capsid proteins and the antibody repertoire elicited during natural infection.

Human papillomavirus (HPV) accounts for >600 000 cancer cases and >250 000 deaths globally per annum [1]. Typical of small double-stranded DNA viruses, HPV exhibits a low evolutionary rate, although distinct genotypes have arisen over time. Whole genome sequencing has classified subgenotype variants into distinct lineages and sublineages [2], thereby improving appreciation of the underlying mechanisms driving genome diversity, disease attribution, and phylogeographic distribution. HPV lineages emerged between 200,000 and 500,000 years ago and sublineages 50,000 and 200,000 years ago, around the time of early modern humans [3]. However, the global diversity of HPV variants and the impact of this variation on the structure and function of individual HPV proteins are unclear.

The HPV capsid comprises 360 copies of the major capsid protein, L1, arranged as an icosahedral lattice of 72 pentamers, while the associated minor capsid protein, L2, facilitates virus infectivity [4]. Surface-exposed L1 loops are the target for neutralizing antibodies, and L1 virus-like particles (VLPs) form the basis of the licensed HPV vaccines [5]. Natural HPV infection can persist for several months before being cleared by host cell–mediated immunity [1], and although serum antibodies are of low titer, they nevertheless may reduce the risk of new infections [6]. Most antibodies elicited following viral infections are nonneutralizing, and this may be a feature of the antibody repertoire following HPV infection. A predominantly low-avidity, nonneutralizing phenotype can be transformed into a high-titer, neutralizing phenotype following vaccination [7], and natural infection antibody specificities may be more heterogeneous than those elicited by vaccination.

We demonstrated that lineage-specific sequence variation in the surface-exposed loops on the L1 protein affects recognition by neutralizing antibodies elicited following natural infection [8]. Here we used the same panel of serum collected from women residing in Africa, the Americas, Asia, and Europe following natural infection with lineage-specific variants of vaccine-preventable oncogenic HPV types (HPV-16, 18, 31, 33, 45, 52, and 58). We compared lineage-specific antigenicity profiles using antibody binding and neutralizing antibody data. These data further delineate the antigenic properties of several oncogenic HPV genotypes and improve our understanding of lineage-specific immunity in natural infection.

## METHODS

### Samples

Serum or plasma samples (n = 2255) representing geographically diverse (Supplementary Figure 1) lineage-specific natural infections were from archives held at the International Agency for Research on Cancer (IARC) and the National Cancer Institute (NCI), the details of which have previously been reported [8].

### Multiplex Binding Assay

Antibody binding to lineage-specific L1L2 VLPs [8] was evaluated in a modified multiplexed ligand-binding assay [9], where type-specific master mixes contained lineage-specific VLP-coupled beads. Samples were screened against all lineage antigens representing that genotype, and positive samples were titrated against all lineage antigens, with outcomes reported as midpoint binding titers. Some samples were retested for quality assurance purposes; for the initial and repeat tests, the median natural log titer ratio was 0.99 (IQR, 0.94–1.00; n = 204).

### Antigenic Clustering Analyses

Hierarchical clustering (https://www.hiv.lanl.gov/content/sequence/HEATMAP/heatmap.html) was conducted on the natural log-transformed binding antibody titer data, with serologic and antigen dendrograms constructed from the resulting euclidean distance matrices and clusters supported by bootstrap values from 500 pseudo-replicates.

The spatial relationship between the lineage antigens was evaluated with antigenic cartography (https://acorg.github.io/Racmacs/) by using binding data generated in this study and published neutralizing antibody data [8]. Antigenic maps were generated in 2 dimensions through 100 optimizations. Negative titers (<50) were censored at 25 for calculation purposes. In each map, a gray grid square represents 1 antigenic unit and is equivalent to a 2-fold distance. To test robustness of the antigenic distance estimates, we generated (1) 10 resampling-with-replacement pseudo-datasets (target: 50 samples per lineage) while maintaining the structure of the original dataset [8] and (2) antigenic maps encompassing 1 SD of the positional variation of the resolved coordinates following 100 bootstrap iterations. For comparison, relational antigenic maps were merged via the Procrustes function in Racmacs.

### Sequence Analysis

Amino acid sequence analysis of the antigens [8] used in this study was performed in MEGA version 11.0 (https://www.megasoftware.net/). Type-specific L1 pentamer crystal models represented HPV-16 (Protein Data Bank, https://www.rcsb.org/; accession 2R5H), HPV-18 (2R5I), HPV-31 (HPV-16 2R5H as closest alternative), HPV-33 (6IGE), HPV-45 (HPV-18 2R5I as closest alternative), HPV-52 (6IGF), and HPV-58 (5Y9E). The MolStar viewer (https://molstar.org/) was used to highlight specific variant residues between the reference (lineage A) and another lineage within each genotype.

### Statistical Analysis

For lineage antigens to be considered antigenically distinct, we set an a priori 4-fold [8] distance threshold between the comparison antigen and lineage A using antigenic cartography. Differences in seropositivity rates (χ^2^ test) and binding antibody titers (Wilcoxon signed rank test) were assessed by the indicated test. Tests were 2-tailed and conducted in Stata version 18.0 SE (StataCorp).

## RESULTS

Lineage-specific seropositivity rates and geometric mean titers (95% CI) are summarized in Supplementary Table 1.

Hierarchical clustering was carried out to resolve the antigenic relatedness of lineage-specific antigens by evaluating the binding antibody responses against these antigens. For each type, lineage antigens were identified as distinct branches within the antigenic dendrograms, with a high degree of bootstrap support (Supplementary Figure 2).

Antigenic cartography was employed to resolve the spatial relationship of the lineage-specific antigens based on their antigenic profiles derived from neutralizing [8] or binding antibody datasets (Supplementary Figure 3). The antigenic profiles derived were geometrically similar, with some minor differences in the magnitudes of the estimated distances (Table 1). For example, HPV-16 lineage C exhibited an antigenic distance approximately 2.7-fold from lineage A antigen, and this was consistent between the datasets. Uncertainty in the derived spatial coordinates was addressed by the generation of bootstrapped antigenic maps incorporating a 1-SD uncertainty margin for each antigen-specific estimate (Supplementary Figure 4) and a resampling with a replacement approach to generate a 95% CI around each distance estimate (Table 1, Supplementary Figure 5). Together, these analyses suggest that the antigenic distance estimates between antigens were robust.

**Table 1.** Antigenic Distance Estimates for Neutralizing and Binding Antibodies

	Neutralizing Antibody Estimated Fold Distance Between Lineages (95% CI)			Binding Antibody Estimated Fold Distance Between Lineages (95% CI)		
HPV: Lineage	B	C	D	B	C	D
HPV-16						
A	1.2 (1.2–1.3)	**2.7** (2.6–2.7)	1.9 (1.9–2.0)	1.2 (1.2–1.2)	**2.7** (2.6–2.8)	1.9 (1.8–1.9)
B		**2.6** (2.5–2.6)	1.7 (1.6–1.8)		**2.4** (2.3–2.5)	1.6 (1.5–1.6)
C			**2.0** (1.9–2.0)			**2.1** (2.0–2.1)
HPV-18						
A	1.3 (1.3–1.3)	1.4 (1.3–1.4)		1.5 (1.4–1.5)	**2.1** (2.0–2.1)	
B		1.3 (1.3–1.4)			**2.3** (2.2–2.3)	
HPV-31						
A	1.3 (1.2–1.3)	1.6 (1.6–1.6)		1.4 (1.4–1.5)	1.9 (1.8–1.9)	
B		1.4 (1.4–1.5)			1.4 (1.4–1.5)	
HPV-33						
A	**4.1** (3.9–4.2)	**5.5** (5.5–5.8)		**4.9** (4.8–5.3)	**5.5** (5.4–5.9)	
B		1.7 (1.7–1.8)			1.2 (1.2–1.3)	
HPV-45						
A	1.8 (1.7–1.8)			**3.8** (3.4–3.9)		
HPV-52						
A	**2.3** (2.2–2.4)	1.1 (1.1–1.1)	**3.8** (3.8–4.1)	**3.0** (2.8–3.1)	**2.2** (2.1–2.3)	**17.1** (16.0–18.2)
B		**2.2** (2.1–2.4)	**3.5** (3.4–3.9)		1.4 (1.3–1.4)	**7.5** (6.5–8.1)
C			**3.6** (3.6–3.8)			**8.9** (7.9–9.6)
HPV-58						
A	1.7 (1.6–1.7)	**10.9** (10.2–11.4)	1.1 (1.1–1.2)	**2.5** (2.3–2.6)	**29.0** (25.0–30.8)	1.6 (1.5–1.6)
B		**10.9** (9.9–11.9)	1.5 (1.4–1.6)		**17.8** (15.4–18.7)	1.6 (1.6–1.7)
C			**11.7** (11.4–12.4)			**20.6** (19.9–22.1)

Abbreviation: HPV, human papillomavirus.

Antigenic distances given in fold differences between indicated lineage antigens (where a 2-fold difference is equivalent to 1 antigenic unit) based on the neutralizing or binding antibody datasets. Bold type indicates differential antigenicity (≥2-fold) between indicated lineage antigens, with distances ≥4-fold being considered antigenically distinct. 95% CIs are from sampling with replacement iterations.

**Figure 1.**
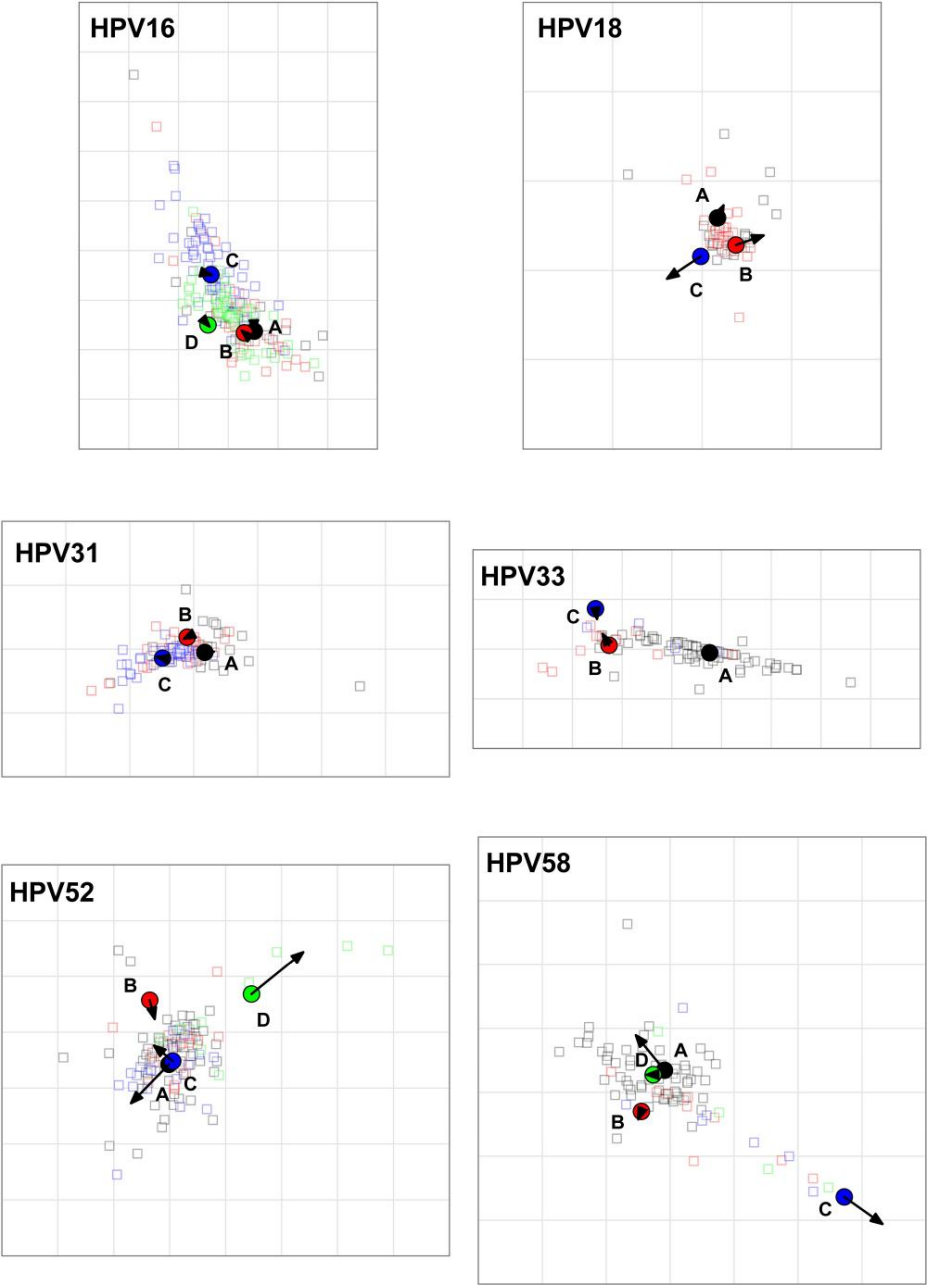
Comparison of relational antigenic distances estimated between neutralizing and binding antibody datasets. Neutralizing and binding antibody titers were subjected to 2-dimensional clustering via antigenic cartography (https://acorg.github.io/Racmacs/) and antigens were aligned with the Procrustes function. Filled-in circles and open squares represent lineage A (black), B (red), C (blue), and D (green) antigens and antibodies, respectively. Arrows indicate geometric shift between the antigen coordinates estimated from neutralizing antibody data and those estimated from the binding antibody data. In each antigenic map regardless of actual size, the gray grid squares represent 1 antigenic unit, which is equivalent to a 2-fold distance between antigens; thus, 3 grid squares are equivalent to an 8-fold distance. While individual serum samples are derived from specific lineage infections and expected to coalesce around their antigens, there is no expectation of strict lineage antibody specificity, as sera will likely display a range of individual antibody specificities and may in some cases be mapped closer to a heterologous antigen than the corresponding homologous antigen. HPV, human papillomavirus.

The antigenic distance maps were subjected to merging (Procrustes analysis) to evaluate the geometric similarity between the relative antigen coordinates estimated for the neutralizing and binding antibody datasets (Figure 1). In most cases, either the estimated coordinates were similar between the datasets (HPV-16, HPV-31, HPV-33), or differences already apparent from the neutralizing antibody dataset were amplified by using the binding antibody dataset (eg, HPV-52 and HPV-58; Table 1). In the case of HPV-18, the binding antibody data demonstrated a ≥2-fold difference between lineage C and the lineage A and B antigens not apparent in the neutralizing antibody dataset, resulting in a small geometrical shift of the lineage C antigen relative to lineage A and B antigens. HPV-45 comprises only 2 lineages (A and B), which limited the accuracy of the estimates of spatial distance between antigens, and outcomes are presented separately (Supplementary Figure 6).

Phylogenetic analysis of amino acid sequence data was insufficient to predict their antigenic relatedness in many cases (compare Supplementary Figures 2 and 7). Variant amino acid residues between the lineage A antigen and a more antigenically distant antigen were mapped onto resolved type-specific L1 pentamer crystals, where available, or the crystal with the closest sequence match (Supplementary Figure 8). For most genotypes, differences in lineage antigenicity were associated with few variant residues. HPV-16 lineage C displayed a 2- to 3-fold distance from lineage A, with 3 residues in the EF (T176N, N181T) and FG (S282P) implicated. HPV-18 lineage C differed from lineage A by a single BC (R51K) loop residue. HPV-31 did not demonstrate any difference (<2-fold) in estimated distance between lineage antigens, suggesting that antigenicity is not significantly affected by variation in 2 FG (T267A and T274N) loop residues. The 5-fold difference in antigenicity between HPV-33 lineages A and B was associated with variant residues in the BC (T56N), DE (G133S), and FG (T266K) loops. HPV-45 lineage B differed from lineage A by residues in the BC (N55S) and HI (G357N) loops. Differences between HPV-52 lineages D and A were mapped to 3 residues in the FG (Q281K) and HI (K354T, S357D) loops. For HPV-58, there were several variant residues in the DE (S133G, P137T), FG (D273N, K266T, A270P, V285G), and HI (G352D, D357N) loops between lineages A and C. These variant residues could be topographically distinct, as in the case of HPV-33 or within a more clustered arrangement such as that for HPV-52.

In many cases, the outlier lineages for a genotype tended to be overrepresented in sequences from Africa (Supplementary Figure 9).

## DISCUSSION

Estimates of the antigenic distances between lineage antigens based on antibody binding were broadly comparable to those derived from neutralizing antibody data, suggesting a degree of similarity between the neutralizing and binding antibody specificities elicited during natural infection [8]. These data also highlighted lineage-specific antigenic profiles not easily predicted by sequence data alone. Thus, resolution of the taxonomic complexity and global dispersal of subgenotype variants should include efforts to better understand the biological implications of such variation.

These antigenicity profiles were comparable to those derived following characterization by neutralizing monoclonal antibodies and nonavalent vaccine sera [10, 11], suggesting a high degree of overlap between these specificities. In addition, HPV-16 lineage C variant residues have been implicated in the footprint of at least 1 neutralizing HPV-16 monoclonal antibody [12], and chimeric HPV-33 antigens have highlighted residues in the BC and DE loops as being important for the binding sites of several MAbs [11, 13]. HPV-52 DE and HI loops are important for recognition by murine antisera, while HPV-58 DE, EF, FG, and HI loops contribute to overall antigenicity [11, 13]. Some of these residues were at or adjacent to those found in other types, suggesting a degree of shared topography of the capsid proteins of related high-risk genotypes. The outlier lineages identified here tend to be overrepresented in sequences from the African region, which contributes to a better understanding of global lineage dispersal during early human migrations [3]. Further study is required to determine whether the lineage-specific antigenicity defined by the natural infection antibody repertoire supports a role for host immunity in the differential global dispersal of lineage variants. Given the similarity of antigenic profiles defined by sera raised to VLP-based vaccines [10], it will be important to monitor the ecologic distribution of lineage variants following the introduction of national vaccine campaigns, particularly in countries with a high prevalence of outlier lineages. The potential impact of lineage variation on vaccine efficacy is starting to be explored in some detail [14].

The implicated domains identified here may overlap with those involved in heparan sulfate receptor binding [15], which would be mechanistically intuitive, though balanced by pressure to remain functionally conserved.

There were shortcomings associated with this study. Although the number of samples included in the panel was significant, the sample collection was not globally representative (Europe, Western Asia, and East Asia were underrepresented), nor were each genotype and lineage equally represented within the panel. Although lineage dispersal is geographically associated, lineage sequences are highly conserved globally, which helps to mitigate against geographic underrepresentation in the sampling. Lineage A was the most common lineage represented, which may have introduced a bias whereby interlineage distances were influenced to a greater extent by the lineage A sera specificity than the non-A specificities. HPV capsids have been used widely to monitor antibody responses to vaccines and natural infection, as well as elucidate the entry process. Although steps are taken to standardize the use of these antigens, including quantification, electron microscopy, and the use of chimeric antigens [8, 9, 11], the contribution of subtle differences in structural integrity to these outcomes cannot be ruled out. However, potential differences between how capsids behave in vitro and how authentic HPVs behave in vivo is a limitation of all antigen-based model systems.

In summary, these data imply that specific and common nonsynonymous mutations found within the HPV L1 capsid gene that have emerged over millennia, concurrent with the rest of the HPV genome, directly affect the antigenicity of the proteins that they encode. These data help to delineate the lineage-specific antigenic relationship among capsid proteins of several oncogenic HPV genotypes and improve our knowledge of capsid topography and the antibody repertoire elicited during natural infection.

## Supplementary Material

jiaf502_Supplementary_Data

## Supplementary Data

Supplementary materials are available at *The Journal of Infectious Diseases* online (http://jid.oxfordjournals.org/). Supplementary materials consist of data provided by the author that are published to benefit the reader. The posted materials are not copyedited. The contents of all supplementary data are the sole responsibility of the authors. Questions or messages regarding errors should be addressed to the author.
