# Supplementary material for: Profiling Human Papillomavirus Lineage-Specific Capsid Antigenicity With Geographically Diverse Natural Infection Antibodies
Source: J Infect Dis. 2025 Oct 1;233(1):e145–50. doi: 10.1093/infdis/jiaf502 (PMC12811858; doi:10.1093/infdis/jiaf502)
Supplement: jiaf502_Supplementary_Data [file jiaf502_supplementary_data.pdf]

Supplementary Table 1. *Binding titers against individual lineage antigens*

HPV	Lineage	n	N	Lineage-specific GMT (95%CI) A	B	C	D
HPV16	A	24	150	509 (297 – 872)	558 (329 – 948)	446 (223 – 891)	329 (168 – 645)
					0.107	*0.623*	*0.143*
	B	33	112	673 (330 – 1373)	824 (409 – 1664)	473 (222 – 1008)	819 (383 – 1751)
				<0.001		***0.004***	***0.038***
	C	35	156	223 (125 – 397)	286 (165 – 495)	499 (289 – 860)	288 (161 – 515)
				<0.001	***0.006***		*0.093*
	D	30	154	621 (382 – 1009)	758 (482 – 1192)	561 (365 – 863)	822 (505 – 1338)
				0.109	*0.503*	***0.006***	
HPV18	A	19	197	239 (139 – 412)	218 (127 – 371)	128 (67 – 244)	
					0.249	***<0.001***	
	B	15	126	97 (63 – 147)	90 (58 – 142)	63 (36 – 108)	
				0.804		*0.289*	
	C	0	4				
HPV31	A	32	148	525 (349 – 792)	503 (330 – 767)	527 (353 – 788)	
					0.645	*0.775*	
	B	29	145	181 (111 – 294)	273 (187 – 399)	308 (214 – 442)	
				<0.001		*0.247*	
	C	28	138	155 (107 – 225)	208 (147 – 295)	324 (254 – 413)	
				<0.001	***<0.001***		
HPV33	A	53	149	196 (126 – 306)	283 (186 – 432)	283 (189 – 425)	
					0.184	*0.374*	
	B	7	28	87 (28 – 268)	608 (166 – 2233)	571 (170 – 1919)	
				0.047		*1.000*	
	C	4	12	50 (12 – 211)	331 (62 – 1777)	377 (75 – 1898)	
				0.250	*0.625*		
HPV45	A	7	150	225 (81 – 625)	63 (28 – 145)		
					0.047		
	B	6	149	103 (31 – 344)	91 (35 – 239)		
				1.000			
HPV52	A	32	146	607 (376 – 981)	326 (210 – 504)	428 (295 – 620)	51 (33 – 80)
					<0.001	***<0.001***	***<0.001***
	B	6	35	634 (338 – 1190)	220 (57 – 848)	245 (63 – 947)	87 (17 – 3430)
				0.156		*0.375*	*0.063*
	C	8	38	648 (139 – 3023)	257 (75 – 881)	391 (138 – 1114)	49 (15 – 161)
				0.016	***0.008***		***0.016***
	D	4	16	368 (21 – 6476)	105 (12 – 930)	181 (17 – 1961)	43 (7 – 253)
				0.250	*0.625*	*0.375*	
HPV58	A	42	149	380 (232 – 622)	395 (247 – 630)	29 (24 – 35)	705 (475 – 1047)
					0.109	***<0.001***	***<0.001***
	B	4	13	216 (10 – 4536)	384 (11 – 12965)	79 (8 – 759)	368 (12 – 11323)
				0.250		*0.250*	*0.750*
	C	4	18	50 (13 – 201)	106 (22 – 497)	90 (7 – 1095)	69 (10 – 449)
				0.625	*0.875*		*0.875*
	D	3	19	120 (4 – 3845)	131 (3 – 5439)	259 (1 – 74379)	168 (3 – 11100)
				0.500	*1.000*	*0.750*	

Number of seropositive positive samples (n) out of total samples (N) representing indicated lineage. GMT (95%CI), geometric mean antibody binding titers. Seropositivity rates of lineages within a genotype were similar (p>0.05; Chi2) for all types. Differences in antibody titers between the indicated lineage antigen associated with infection from which the serum was derived and the remaining antigens highlighted in bold type (Wilcoxon paired sign test). Three HPV16 samples were excluded due to poor sample quality.

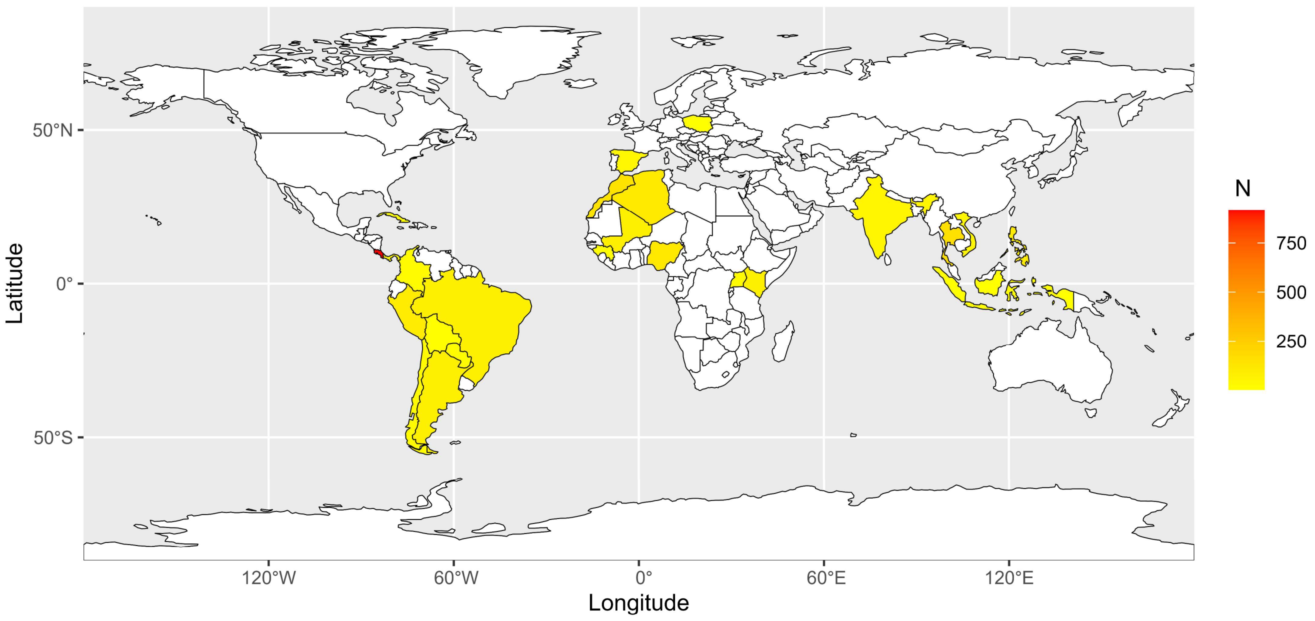

Supplementary Figure 1. *Geographical distribution of sample collection*.
Global map represented by a heatmap with indicative scale for sample collection distribution. Map image generated using R (R Foundation for Statistical Computing, Vienna, Austria; https://www.R-project.org/), world map JSON file (https://github.com/AshKyd/geojson-regions) and ggplot2 (Wickham H 2016. ggplot2: Elegant Graphics for Data Analysis; Springer-Verlag New York https://ggplot2.tidyverse.org).

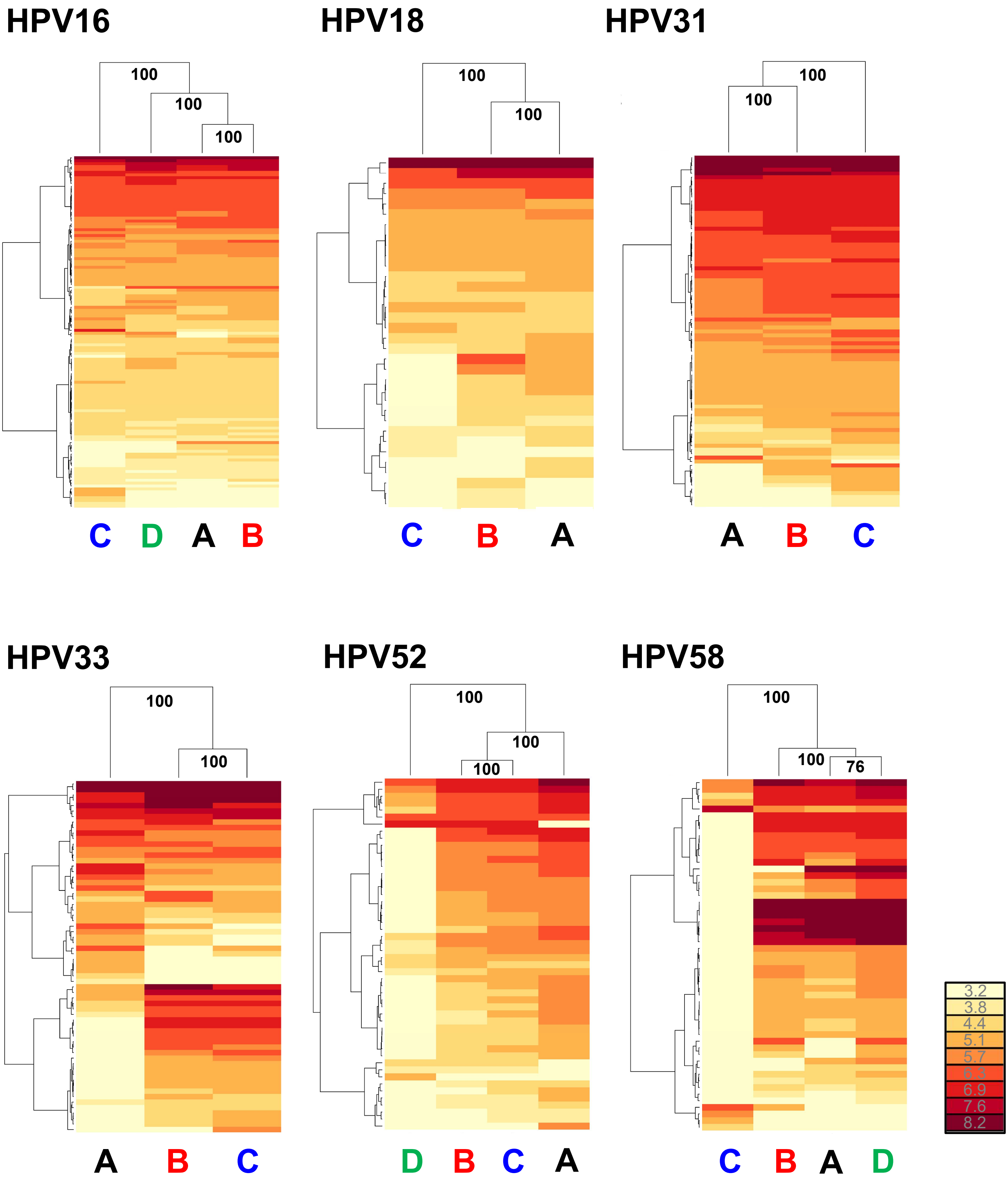

Supplementary Figure 2. *Heatmaps of binding antibody responses*
Antibody titers were natural log-transformed and subjected to two-dimensional hierarchical clustering with resulting data re-ordered according to serological (left) and antigen (top) dendrograms constructed from the resulting Euclidean distance matrices with the antigen clusters supported by the indicated percentage of 500 bootstrapped pseudoreplicates (https://www.hiv.lanl.gov/content/sequence/HEATMAP/heatmap.html). Lineage-specific antigens labelled at the base of the heatmap (A, black; B, red; C, blue; D, green). The antigen clusters are supported by data from many sera whereas the serum clusters are supported by data from relatively few antigens; thus, more weight should be given to the definition of antigen clusters that have bootstrap support. An indicative natural log heatmap scale bar is shown.

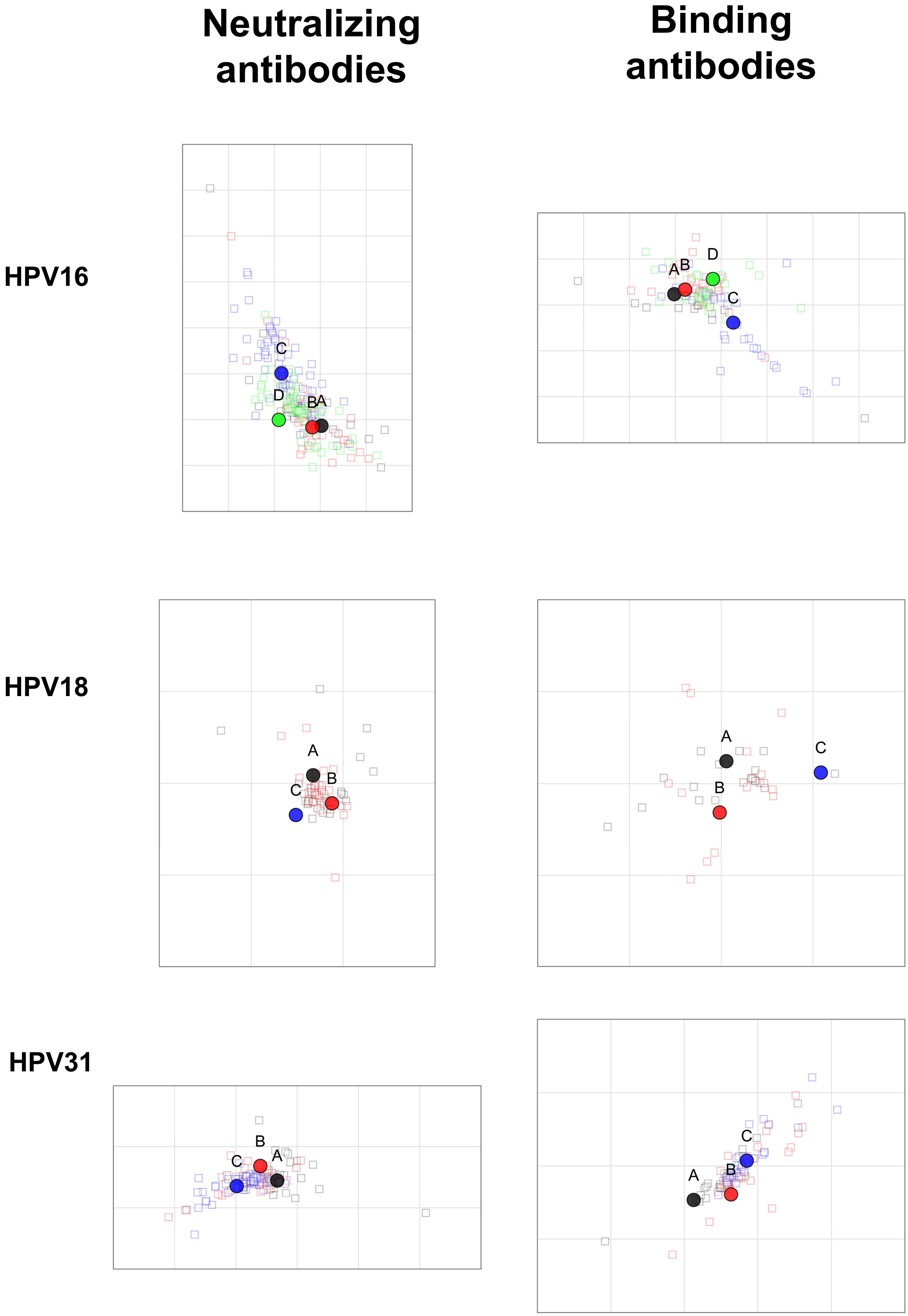
Neutralizing antibodies
Binding antibodies
HPV16
HPV18
HPV31
A
B
C
D

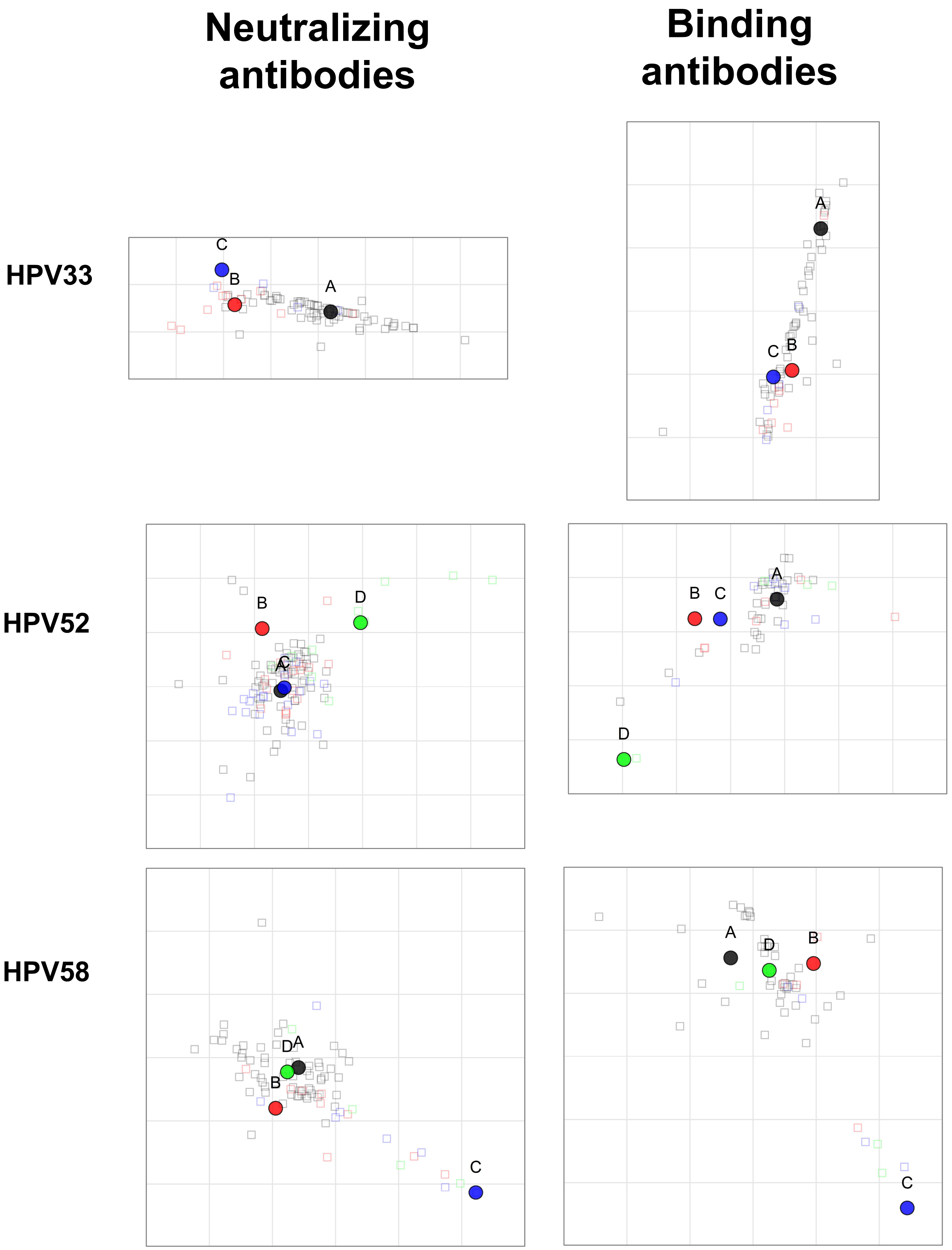

Supplementary Figure 3. *Antigenic maps of neutralizing and binding antibody responses*
Neutralizing and binding antibody titers were subjected to two-dimensional clustering using the antigenic cartography package (https://acorg.github.io/Racmacs/). Filled in circles and open squares represent lineage A (black), B (red), C (blue), and D (green) antigens and antibodies, respectively. In each antigenic map regardless of actual size, the grey grid squares represent 1 antigenic unit (AU), which is equivalent to a 2-fold distance between antigens; thus, three grid squares are equivalent to an 8-fold distance. While individual serum samples are derived from specific lineage infections and expected to coalesce around their respective antigen, there is no expectation of a strict lineage antibody specificity as sera will likely display a range of antibody specificities and may in some cases be mapped closer to a heterologous antigen than the corresponding homologous antigen.

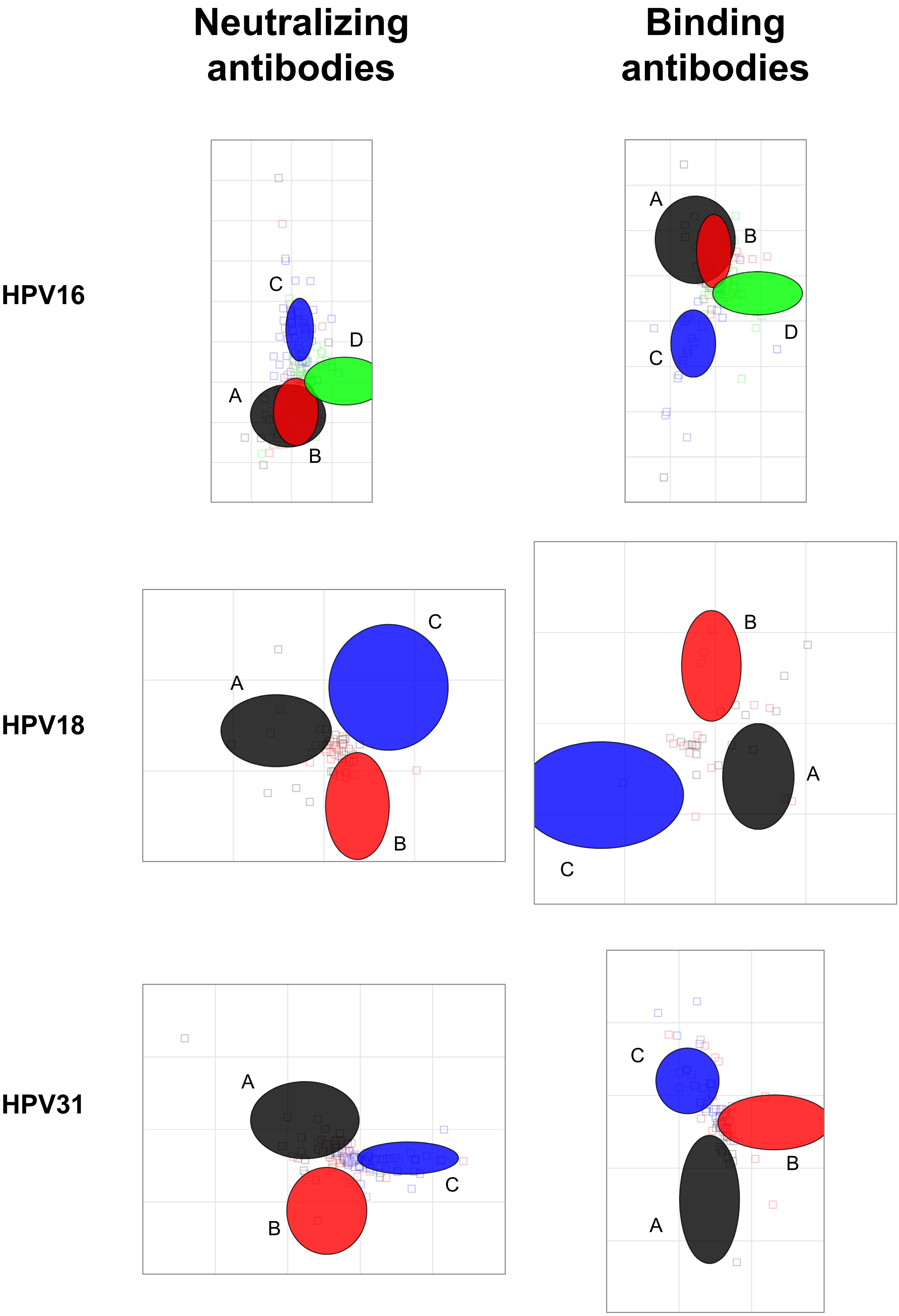

Neutralizing antibodies
Binding antibodies
HPV16
HPV18
HPV31
A
B
C
D

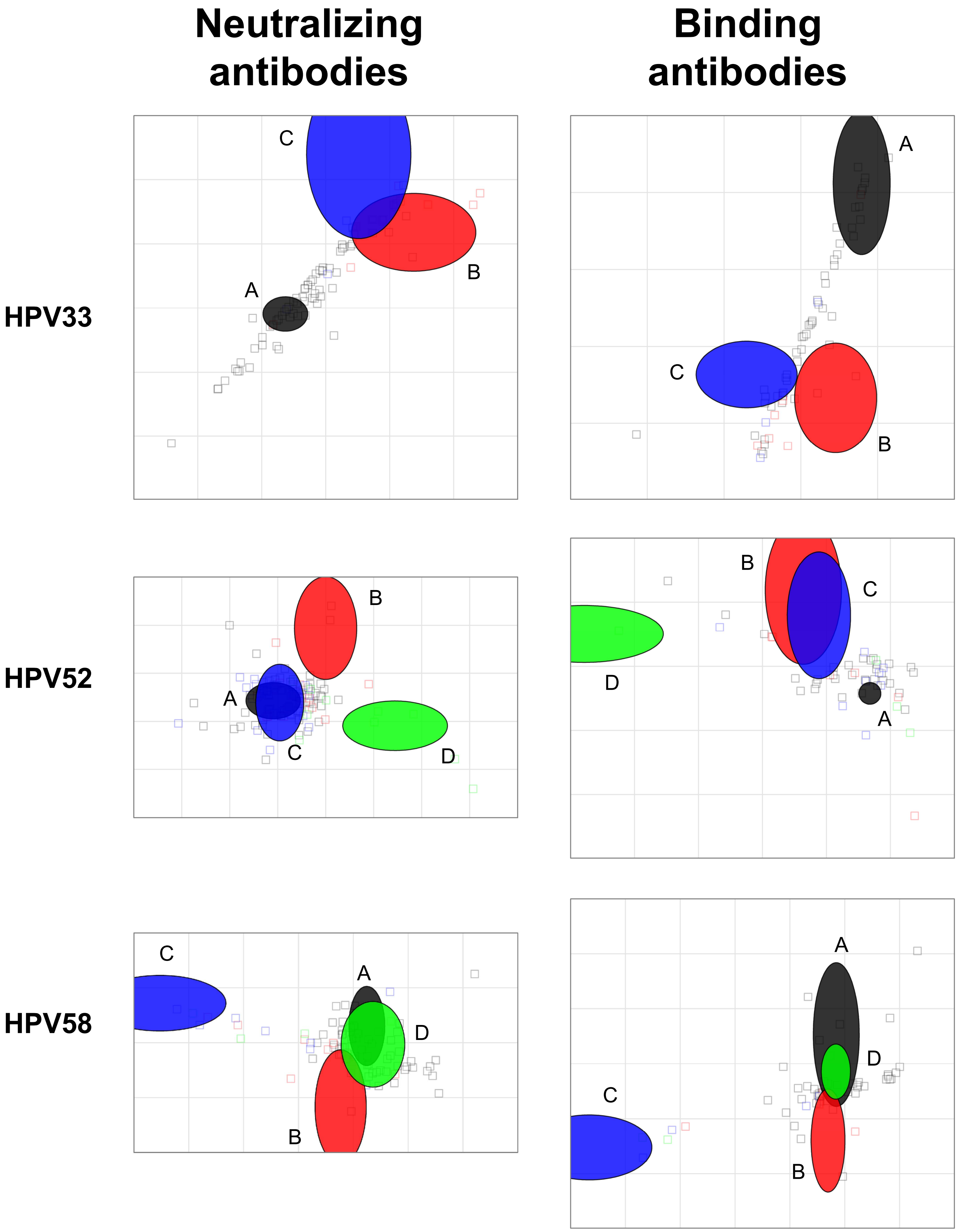

Supplementary Figure 4. *Bootstrapped antigenic maps including geometric uncertainty around antigen positions.* Neutralizing and binding antibody titers were subjected to two-dimensional clustering using the antigenic cartography package (https://acorg.github.io/Racmacs/). Filled in circles and open squares represent lineage A (black), B (red), C (blue), and D (green) antigens and antibodies, respectively. In each antigenic map regardless of actual size, the grey grid squares represent 1 antigenic unit (AU), which is equivalent to a 2-fold distance between antigens; thus, three grid squares are equivalent to an 8-fold distance. Each oval region indicates the area in which 68% (one standard deviation; 1SD) of the positional variation of an antigen is captured.

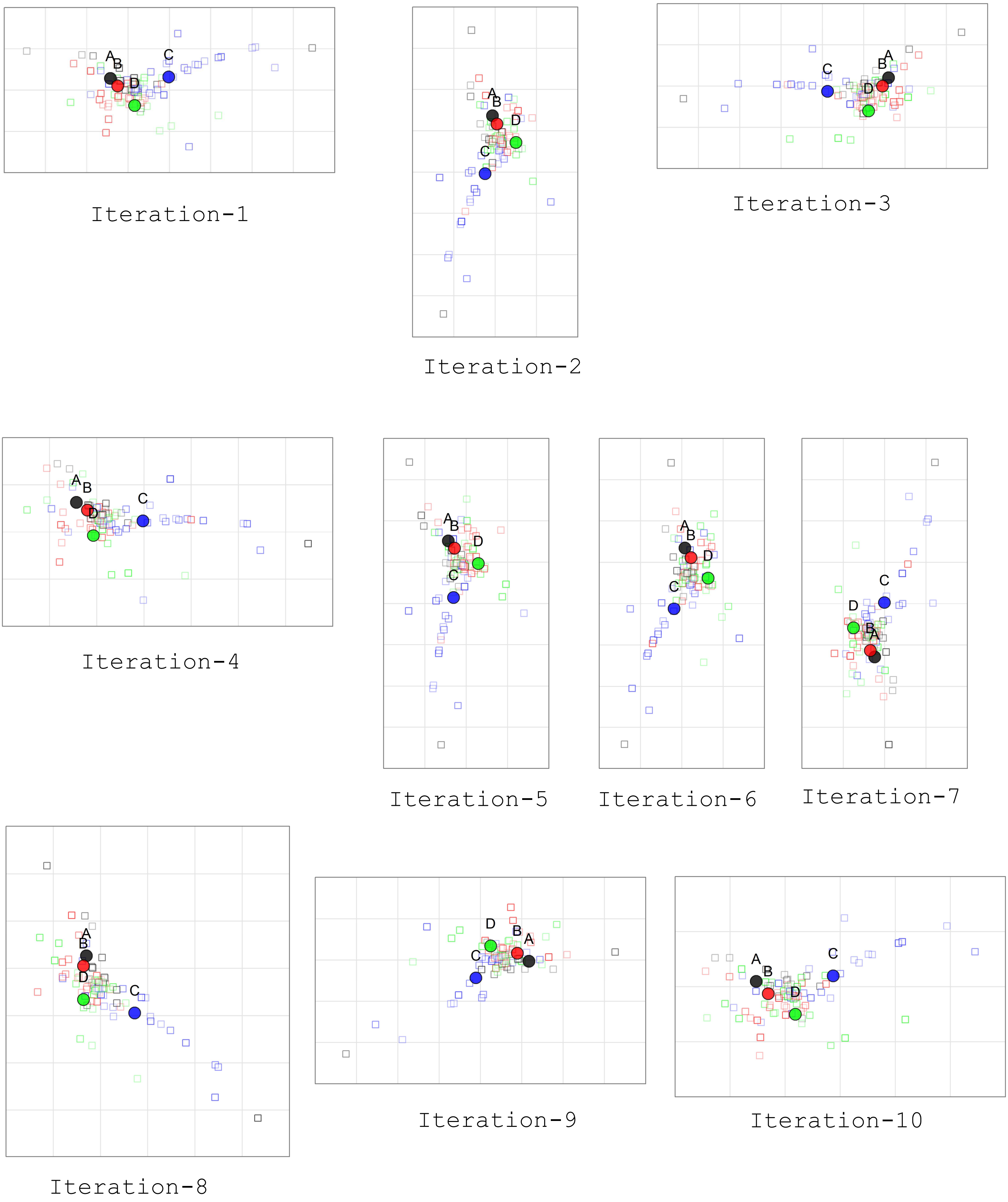

Supplementary Figure 5. *Antigenic resampling with replacement*
To test the robustness of the antigenic mapping for each genotype a resampling with replacement method was employed. The dataset for each genotype was subjected to 10 such randomizations (iterations) creating 10 pseudo-replicate antigenic maps. An example using HPV16 is shown with lineages coded as follows: A, black; B, red; C, blue; D, green. The 95% confidence of these estimates is provided in **Table 1**.

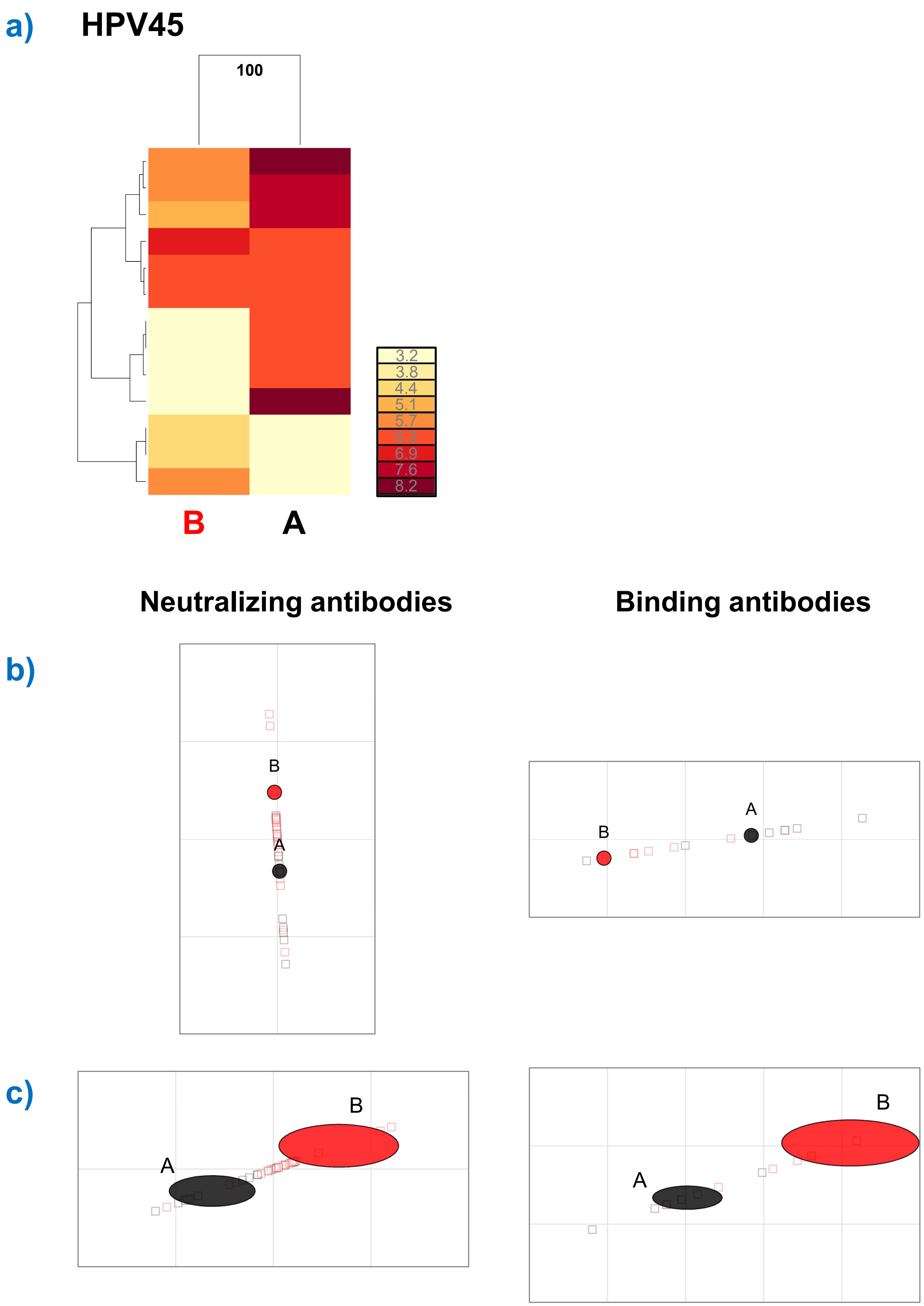

Supplementary Figure 6. *HPV45 antigenic clustering analysis*

(a) Two-dimensional hierarchical clustering with serological (left) and antigen (top) dendrograms constructed from the resulting Euclidean distance matrices with the antigen clusters supported 100% of 500 bootstrapped pseudoreplicates. Lineage-specific antigens labelled at the base of the heatmap (A, black; B, red). (b) Antibody titers were subjected to two-dimensional clustering using the antigenic cartography package (https://acorg.github.io/Racmacs/). Filled in circles and open squares represent antigens and antibodies, respectively. In each antigenic map regardless of actual size, the grey grid squares represent 1 antigenic unit (AU), which is equivalent to a 2-fold distance between antigens. (c) Each oval region indicates the area in which 68% (one standard deviation; 1SD) of the positional variation of an antigen is captured.

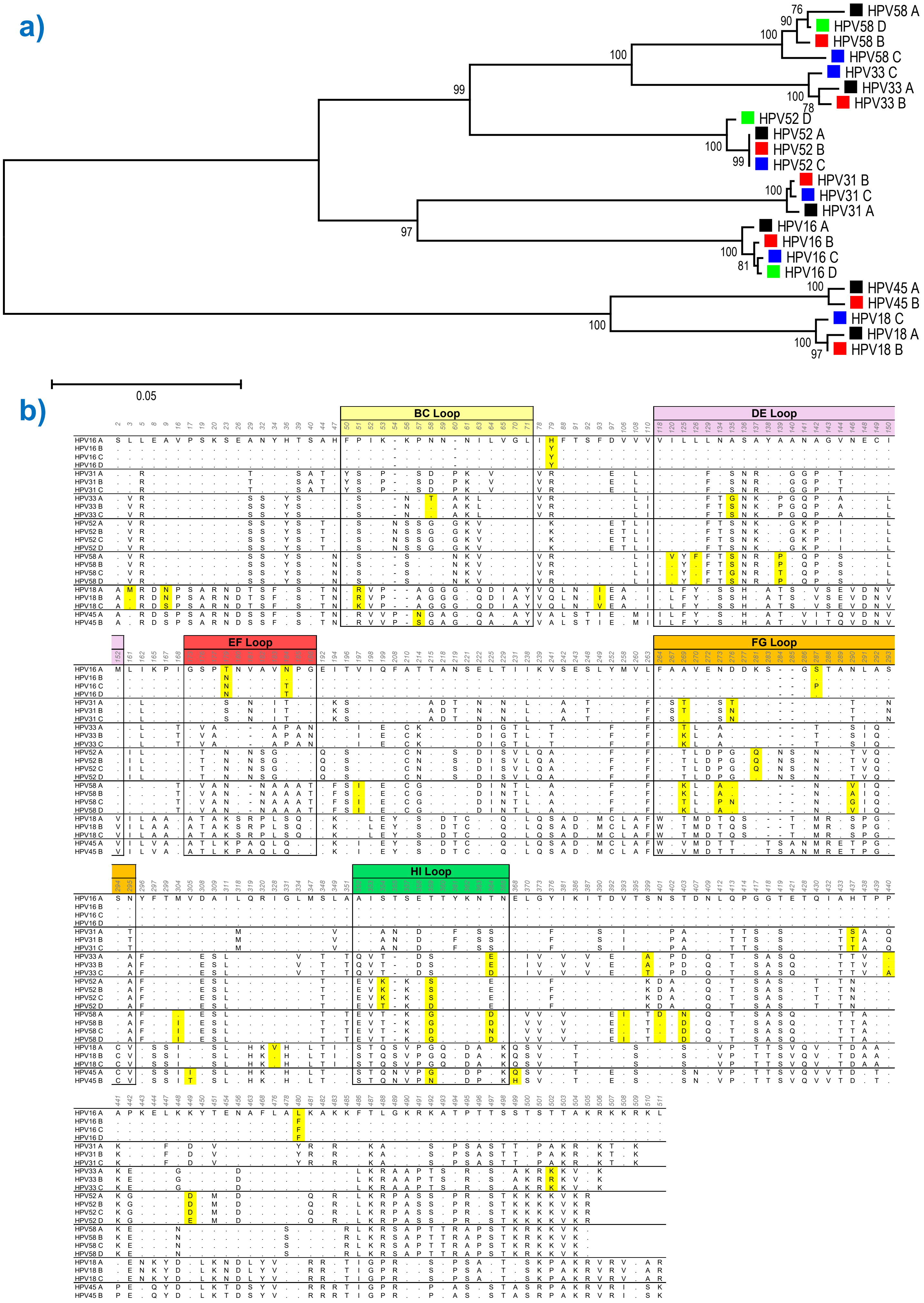

Supplementary Figure 7. *HPV L1 sequence variation between antigens used in this study*

(a) Phylogenetic tree constructed from L1 amino acid sequences representing lineages A (black squares), B (red), C (blue) and D (green) antigens using the Neighbor-Joining algorithm (500 bootstrap replicates, with branches ≥70% support indicated) (https://www.megasoftware.net/). (b) Alignment of lineage-specific L1 sequences with variable residues shown and intra-type polymorphic residues highlighted. Position of the external loops BC, DE, EF, FG and HI indicated.

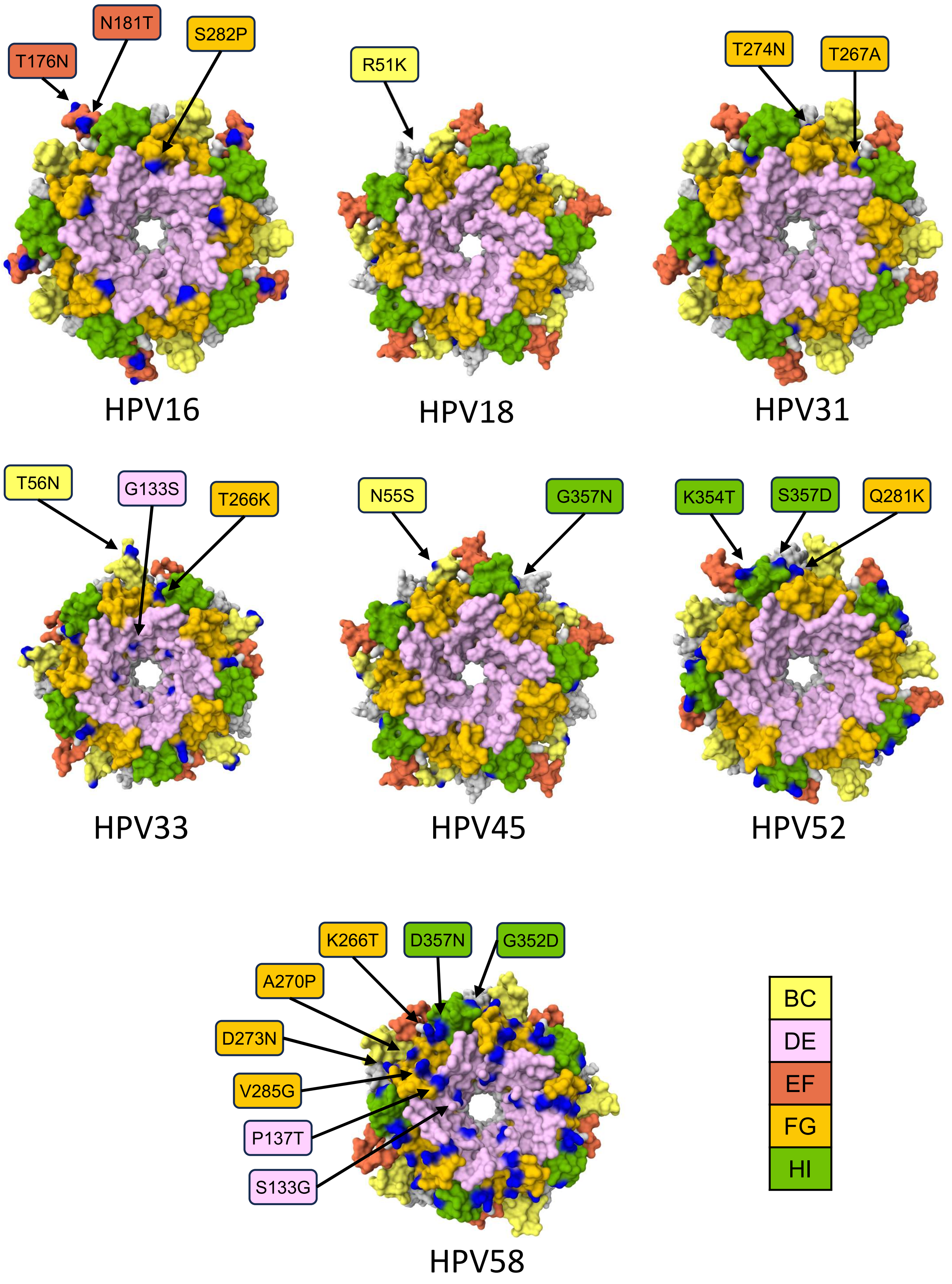

Supplementary Figure 8. *Mapping of lineage-specific amino acid polymorphisms*
HPV L1 pentamer crystals models of HPV16 (https://www.rcsb.org/ PDB accession number 2R5H), HPV18 (2R5I), HPV31 (HPV16 2R5H), HPV33 (6IGE), HPV45 (HPV18 2R5I), HPV52 (6IGF) and HPV58 (5Y9E) with top view shown (https://molstar.org/). External surface loops are shaded as indicated (BC, yellow; DE, pink; EF, red; FG, orange; HI, green). Surface exposed amino acid polymorphisms between lineage A sequence and the indicated comparison sequence for HPV16 (lineage C), HPV18 (lineage C), HPV31 (lineage C), HPV33 (lineage B and common sites in lineage C), HPV45 (lineage B), HPV52 (lineage D) and HPV58 (lineage C) are indicated in blue.

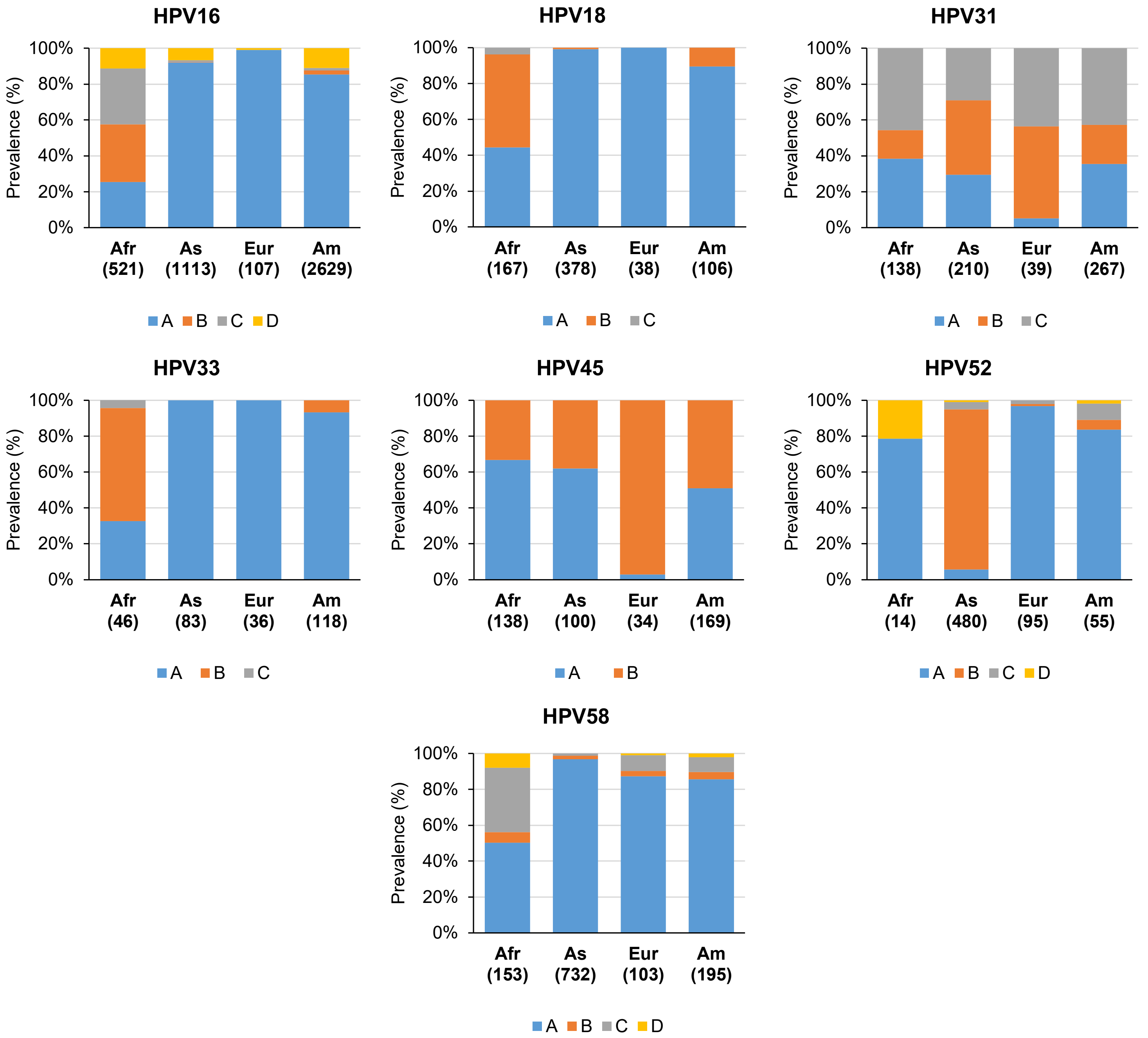

Supplementary Figure 9. *Estimates of global type-specific lineage distribution*
Geographical region for source of sequences as indicated (Afr, Africa; As, Asia; Eur, Europe and Am, The Americas) with the number of collated lineage sequences in parentheses. Sequences were derived from fragment or whole genome sequencing as indicated in the source material: HPV16 (references 1-3, below), HPV18 (4), HPV31 (5-6), HPV33 (6-7), HPV45 (6,8), HPV52 (9-10) and HPV58 (10-12).

1. Clifford et al., Human papillomavirus 16 sub-lineage dispersal and cervical cancer risk worldwide: Whole viral genome sequences from 7116 HPV16-positive women. Papillomavirus Res. 2019 Jun;7:67-74
2. Le et al., Distribution of human papillomavirus among Vietnamese women with cervical cancer and unusual genetic variability of HPV16. Virology. 2024 Jun;594:110058
3. Rader et al., Genetic variations in human papillomavirus and cervical cancer outcomes. Int J Cancer. 2019 May 1;144(9):2206-2214
4. Chen et al., Human Papillomavirus 18 Genetic Variation and Cervical Cancer Risk Worldwide. J Virol. 2015 Oct;89(20):10680-7
5. Pinheiro et al., Phylogenomic Analysis of Human Papillomavirus Type 31 and Cervical Carcinogenesis: A Study of 2093 Viral Genomes. Viruses. 2021 Sep 28;13(10):1948
6. Shing et al., Differential long-term bivalent HPV vaccine cross-protection by variants in the Costa Rica HPV vaccine trial. NPJ Vaccines. 2024 Jun 8;9(1):101
7. Chen et al., Human papillomavirus 33 worldwide genetic variation and associated risk of cervical cancer. Virology. 2014 Jan 5;448:356-62
8. Koestler et al., Whole-genome sequencing of 1,083 HPV45 cases and controls identifies genetic variants associated with glandular cervical lesions. Int J Cancer. 2025 May 5. doi: 10.1002/ijc.35464
9. Zhang et al., Geographical distribution and risk association of human papillomavirus genotype 52-variant lineages. J Infect Dis. 2014 Nov 15;210(10):1600-4
10. Tenjimbayashi et al., Whole-genome analysis of human papillomavirus genotypes 52 and 58 isolated from Japanese women with cervical intraepithelial neoplasia and invasive cervical cancer. Infect Agent Cancer. 2017 Aug 4;12:44
11. Chen et al., Ancient Evolution and Dispersion of Human Papillomavirus 58 Variants. J Virol. 2017 Oct 13;91(21):e01285-17
12. Chan et al., Identification of human papillomavirus type 58 lineages and the distribution worldwide. J Infect Dis. 2011 Jun 1;203(11):1565-73
